# Protocol for a systematic review and network meta-analysis of the use of prophylactic antibiotics in hand trauma surgery

**DOI:** 10.1186/s13643-024-02573-6

**Published:** 2024-06-14

**Authors:** Chen Zhang, Suraya Mohamed Yusuf, Soma Farag, Ryckie George Wade, Justin Conrad Rosen Wormald

**Affiliations:** 1 Oxford University Hospitals NHS Foundation Trust, John Radcliffe Hospital, Headley Way, Headington, Oxford, Oxfordshire OX3 9DU UK; 2 Leeds Institute for Medical Research, University of Leeds, Leeds, UK; 3 Department of Orthopaedics, Rheumatology and Musculoskeletal Sciences, Kadoorie Centre, Oxford Trauma and Emergency Care, Nuffield University of Oxford, Oxford, OX3 9DU UK

**Keywords:** Network meta-analysis, Antibiotic, Hand, Wrist, Surgical site infection

## Abstract

**Background:**

The use of prophylactic antibiotics in surgery is contentious. With the rise in antimicrobial resistance, evidence-based antibiotic use should be followed. This systematic review and network meta-analysis will assess the effectiveness of different antibiotics on the prevention of surgical site infection (SSI) following hand trauma surgery.

**Methods and analysis:**

The databases Embase, MEDLINE, CINAHL and CENTRAL, ClinicalTrials.gov and the WHO International Clinical Trials Registry Platform will be searched. Abstracts will be screened by two persons independently to identify eligible studies.

This systematic review will include both randomised and non-randomised prospective comparative studies in participants with hand and/or wrist injuries requiring surgery; bite injuries will be excluded. The network meta-analysis will compare the use of different prophylactic antibiotics against each other, placebo and/or no antibiotics on the development of SSI within 30 days of surgery (or 90 days if there is an implanted device).

The Cochrane risk-of-bias tool 2 will be used to assess the risk of methodological bias in randomised controlled trials, and the Newcastle-Ottowa scale (NOS) will be used to assess the risk of bias in non-randomised studies.

A random-effects network meta-analysis will be conducted along with subgroup analyses looking at antibiotic timing, injury type, and operation location. Sensitivity analyses including only low risk-of-bias studies will be conducted, and the confidence in the results will be assessed using Confidence in Network Meta‐Analysis (CINEMA).

**Discussion:**

This systematic review and network meta-analysis aims to provide an up-to-date synthesis of the studies assessing the use of antibiotics following hand and wrist trauma to enable evidence-based peri-operative prescribing.

**Systematic review registration:**

PROSPERO CRD42023429618.

**Supplementary Information:**

The online version contains supplementary material available at 10.1186/s13643-024-02573-6.

## Background

Hand injuries account for approximately 20% of accident and emergency attendances [1], and its incidence is increasing [2]. Not only do hand injuries present a significant burden to the health system but also their influence on a patient’s work capacity and daily activities present an additional economic impact [3]. This impact is compounded by complications after hand surgery, including surgical site infections (SSI), resulting in loss of function and poor outcomes [4, 5]. Previous literature shows that the risk of SSI after hand trauma surgery is at least 5–10%, but this may be even higher [6, 7]. Although numerous interventions exist to reduce SSI risk in surgery, few have been tested in randomised controlled trials (RCTs) in hand trauma. Systemic antibiotics are widely used in hand surgery to minimise infectious surgical complications and subsequent morbidity [8]. However, with the rise in global antimicrobial resistance, the efficacy of prophylactic antibiotics should be evaluated to support the judicious use of antibiotics.

A variety of studies have shown the lack of efficacy of prophylactic antibiotic in elective hand surgeries [9]. Since then, further prospective cohort studies have been published by Kistler et al. [10] and Backer et al. [11] looking at 377 and 434 patients respectively undergoing elective hand surgery and again showing no benefit of prophylactic antibiotics in elective hand surgery. The use of prophylactic antibiotics in traumatic hand surgery was explored by Murphy et al. (2016), in patients undergoing surgery for simple hand surgeries [12], and again, they showed no therapeutic benefit of prophylactic antibiotic in reducing the risk of SSI (*RR* 0.89, 95% *CI* 0.65–1.23, restricting to five double-blind RCTs *RR* 0.66, *CI* 0.36–1.21). Both analyses yielded wide 95% confidence intervals, meaning that there is residual uncertainty.

In the proposed network meta-analysis (NMA), we will pool all prospective comparative studies to assess the efficacy of different classes of antibiotics, no antibiotics and placebo in their prevention of post-surgery SSI for hand and wrist injuries. As NMAs assess both direct and indirect evidence, they have several distinct advantages over standard (pairwise) meta-analyses, including better precision and power [13], the ability to compare interventions that have not been directly compared before (i.e. in a real-life head-to-head study) and the capacity to rank competing treatments to inform clinical decisions [14]. This may enable us to generate robust evidence to form the basis of guidelines and inform the future direction of research in relationship to antibiotic use in hand and wrist surgery.

## Method and analysis

This NMA will follow the PRISMA guidelines extension for NMA (see Additional file 1) [15]. This protocol has been registered with PROSPERO (ID CRD42023429618). The report in PROSPERO will be updated with any required amendments.

### Characteristics of studies

All prospective comparative studies comparing active antibiotics, or to placebo or no antibiotic in patients undergoing surgery following hand and/or wrist trauma, will be included. Both randomised and non-randomised trials will be included to increase sample size and increase estimate precision as well as improving network connectivity.

### Characteristics of participants

When screening studies, we will look for participants undergoing hand and/or wrist surgery for traumatic injuries within 2 weeks of their injury. We will not exclude any studies based on patient age, gender, ethnicity, comorbidities or injury severity.

Participants with elective operations for non-traumatic injuries will be excluded. Participants with bite injuries will be excluded due to consideration to the violation of the transitivity assumption, i.e. inclusion of patients with bite injuries introduce interventions in the network for which other participants are not jointly randomizable to. For studies that have included patient with these criteria, we will contact the author for bespoke data cuts according to our inclusion criteria. If such data is not available, the study will be excluded.

### Interventions

All antibiotics in oral or injectable form used within its licensed therapeutic dosages will be included. Antibiotics will be grouped based on their classes, i.e. macrolides, penicillins and cephalosporins reflecting their mechanism of action. Both oral, IM and IV, forms will be grouped together due to their common short-acting nature. Placebo and no antibiotic use will be grouped due to an anticipated lack of placebo effect on SSI development. It has been hypothesised that antibiotics should be given 30–60 min before surgery to allow tissue concentration to reach therapeutic levels at the time of operation [16]. We will thereby assess the effect of the timing of antibiotic use (pre-, intra-, post-operative) with further subgroup analyses.

### Outcome measures

The primary outcome investigated will be a dichotomous outcome assessing the development of surgical site infection within 30 days of the operation or within 90 days if a prosthetic material is implanted (as defined by the CDC) [17]. SSI diagnosis by any method will be included and its definition outlined in a descriptive table.

### Search strategy and study selection

The electronic databases Embase, MEDLINE, CINAHL and CENTRAL will be searched for published comparative studies. The electronic search will be supplemented by a manual search for unpublished and ongoing comparative studies in ClinicalTrials.gov and the WHO International Clinical Trials Registry Platform (ICTRP). We will also perform a manual search of Google Scholar to identify further grey literature. We will use citationchaser to perform forward and backwards citation chasing [18]. We will include all studies irrespective of their publication date, country of origin or language. Studies which are not possible to be translated into English will be excluded from the analysis. There will be a minimum period of 12 months between the last search and submission. Two persons will independently review references and abstracts retrieved by the search to identify eligible studies. Disagreements will be resolved via a discussion with a third member, and a study attrition chart will be used to present the outcomes of the search strategy and subsequent screening process.

### Data extraction

Data will be extracted from the eligible studies and cross-checked for data discrepancies by a second reviewer. Information extracted will include the following:

- General study characteristics (e.g. author, publication year, study type)
- Methodology information (e.g. duration, blinding, randomisation, SSI criteria)
- Participant characteristics (e.g. age, comorbidities, gender)
- Injury characteristics (e.g. type of injury, operation performed, time to surgery)
- Antibiotic characteristics (dose, mode, type, timing of use)
- Outcome measures (SSI development, adverse effects)

The dichotomous primary outcome of SSI will be recorded in the outcome measures section descriptively and as a proportion of overall study population. Completeness of follow-up of 30 days (or 90 days in implanted devices) and attrition analyses will be evaluated in the risk-of-bias assessment. Adverse events will be noted and analyses conducted if sufficient data is extracted.

We anticipate a high variability of definition and determination of SSI as this is a subjective outcome which will be dependent on factors such whether this is reported by a clinician or self-reported by the patient or whether an in-person clinical examination is conducted compared to telephone questionnaires. There will also be variability on other wound management techniques such as irrigation and antiseptic cleaning. These details will be collated from the papers published and presented in a descriptive table. Authors will be contacted to acquire missing data.

### Risk-of-*bias* assessment

The risk of bias will be evaluated in the following domains: allocation sequence, allocation concealment, blinding of participants and study personnel, blinding of outcome assessment, completion of follow-up, selective reporting and other domains including sponsorship bias. The risk of bias of RCTs will be assessed using the Cochrane RoB-2 tool [19], and non-randomised studies will be assessed with the Newcastle–Ottawa scale (NOS) [20]. Risk of bias will be assessed in duplicate by two reviewers and inconsistencies discussed with a third member.

### Data analysis

Transitivity is the fundamental assumption of NMAs and will be investigated carefully as treatments cannot be jointly analysed if the network is intransitive [21]. We assume that patients who fulfil the inclusion criteria are equally likely to receive any of the antibiotic treatments we are planning to compare. Clinical characteristics which have not been shown to affect infection development in hand surgery include location of operation [22], time to surgery [23], depth and extent of injury [24] and diabetes [9]. We will however investigate factors including age, operation location, injury type and time to operation with regard to its distribution between the studies. If the collected studies appear to be sufficiently similar with respect to the distribution of effect modifiers, we will proceed to NMA.

We will produce a network plot to summarise the interventions followed by a series of frequentist, random-effects NMAs using the netmeta package in R assuming a single heterogeneity parameter [25].

To assess the agreement between randomised and non-randomised studies, we will perform separate NMAs and compare the results [26]. This will be supplemented by a series of “designed-adjusted analyses”, whereby data from randomised studies will be combined with down-weighted data from non-randomised studies (NRS) using the following variance inflation factors: *w* = 1 (corresponding to the naïve NMA, i.e. all studies at face value), 0.8, 0.6, 0.4, 0.2 and 0 (i.e. zero excludes NRS). These will be displayed as forest plots per treatments against the reference. If no discrepancies are observed in any of these analyses, we will proceed to joint (“naïve”) analysis pooling both randomised and non-randomised data as the primary analysis.

Interventions will be ranked by their P-scores using the netrank function; P-scores are assumed to take a value between 0 and 1, with a higher score indicating a better treatment [27]. With the netleague package, we will generate league tables with the intervention efficacy ordered by P-score. Forest plots of relative risks (RR) and 95% confidence intervals (CI) will be generated with placebo as the reference treatment. Heterogeneity will be quantified through the standard deviation of random effects (τ, assumed common for all comparisons). To assess inconsistency, we will use both global and local methods with the netsplit package [28, 29] and display the findings via heat plots using the netheat command [30]. In case of inconsistency, we will investigate for possible sources and, if appropriate, further explored by network meta-regression and subgroup analyses.

Given that SSI is rare, we will perform sensitivity fixed-effects Mantel–Haenszel NMA [31] using the *netmetabin* package, and inconsistency will be assessed using the *netsplit* package and SIDDE approach.

Network meta-regressions or subgroup analyses will be used to investigate the impact of (a) injury type, (b) operation location and (c) antibiotic timing. There will likely be heterogeneity and inconsistency due to the wide range of study settings and the relatively small sample size.

We will explore the confidence in estimates of the conclusion which will be evaluated with the Confidence in networked meta-analysis (CINeMA) framework which considers the six domains within-study bias, reporting bias, indirectness, heterogeneity, incoherence and imprecision [32].

To estimate the overall prevalence of SSI, we will use the R package metaprop [33] with Hartung-Knapp-Sidik-Jonkman random-effects and the Freeman-Tukey double arcsine transformation to stabilise the variances.

The relationship between study size and effect size (also known as small study effects) will be explored with a comparison-adjusted funnel plot.

## Discussion

Current NICE guidelines recommend prophylactic antibiotics for clean surgery involving the placement of a prosthesis or implant, clean-contaminated surgery, contaminated surgery and surgery on a dirty or infected wound [34]. No specific guidelines are provided for hand trauma leading to a wide variation in antibiotic use in clinical practice.

Although the meta-analysis published in 2016 [12] showed no difference in the use of antibiotics in preventing SSIs in simple hand injuries, the paper was limited by the low number of robust studies, and the quantitative result was derived from pooling experimental and observational data without a network. On closer inspection of the result, the pooled risk ratio was 0.89 with a wide 95% *CI* 0.65–1.23. In addition, the result of the studies with lower risk of biases Whittaker et al., and Berwald et al., both also have a risk ratio of 0.61 and 0.17 with wide confidence intervals.

Studies assessing complex hand injuries such as fractures and crush injuries present mixed conclusions regarding antibiotic use. Ketonis et al. published a systematic review in 2017 looking at SSIs in open fractures of the hand and concluded the use of antibiotics associated with lower odds of infection [35]. However, the double-blind RCT included within the review conducted by Stevenson et al. in 2003 showed no significant difference in the incidence of SSIs in patients receiving antibiotics compared with a placebo [36]. In addition, the double-blind RCT conducted by Aydin et al. (2010) again showed antibiotics did not significantly affect the SSI incidences in complex hand injuries [37].

Evidence supporting the role of antibiotics in hand trauma is generally poor, with a paucity high-quality randomised studies and predominance of small, single-centre observational studies. Therefore, the aim of this study is to use the power of a NMA to update the current evidence base regarding antibiotic use in all hand trauma and support development of clear guidelines to allow evidence-based antibiotic use in trauma-related hand surgery .

### Supplementary Information

Supplementary Material 1.

Supplementary Material 2. Appendix 1: Search strategy for EMBASE, MEDLINE, CINAHL and CENTRAL. (((((hand) OR (wrist)) OR (finger)) OR (digit)) AND (((antibiotic) OR (antimicrobial)) OR (antibacterial))) AND ((((((injury) OR (wound)) OR (laceration)) OR (trauma)) OR (surgery)) OR (fracture)).

## Data Availability

The datasets used and/or analysed during the current study are available from the corresponding author upon reasonable request.

## Abbreviations

BSSH: British Society for Surgery of the Hand
CDC: Centre for Disease Control and Prevention
CI: Confidence interval
CINeMA: Confidence in Network Meta‐Analysis
NICE: National Institute for Health and Care Excellence
NMA: Network meta‐analysis
NOS: Newcastle-Ottawa scale
PRISMA: Preferred Reporting Items of Systematic Reviews and Meta‐Analyses for Systematic Review Protocols
PROSPERO: International Prospective Register of Systematic Reviews
RCT: Randomised controlled trial
RoB: Risk of bias
SIDDE: Separating indirect evidence from direct evidence
RR: Risk ratio
SSI: Surgical site infection
