# Supplementary material for: Protocol for a systematic review and network meta-analysis of the use of prophylactic antibiotics in hand trauma surgery
Source: Syst Rev. 2024 Jun 14;13:157. doi: 10.1186/s13643-024-02573-6 (PMC11177469; doi:10.1186/s13643-024-02573-6)
Supplement: Supplementary file 2 — Supplementary Material 2. Appendix 1: Search strategy for EMBASE, MEDLINE, CINAHL and CENTRAL. (((((hand) OR (wrist)) OR (finger)) OR (digit)) AND (((antibiotic) OR (antimicrobial)) OR (antibacterial))) AND ((((((injury) OR (wound)) OR (laceration)) OR (trauma)) OR (surgery)) OR (fracture)). [file 13643_2024_2573_MOESM2_ESM.docx]

**Appendix 1**

**Search strategy for EMBASE, MEDLINE, CINAHL and CENTRAL**

(((((hand) OR (wrist)) OR (finger)) OR (digit)) AND (((antibiotic) OR (antimicrobial)) OR (antibacterial))) AND ((((((injury) OR (wound)) OR (laceration)) OR (trauma)) OR (surgery)) OR (fracture))
